# Optimizing Urgent Suspected Colon Cancer Referrals and Reducing Colonoscopy Wait Times in Wales

**DOI:** 10.7759/cureus.76597

**Published:** 2024-12-29

**Authors:** Atreya Subramanian, Ashwin Nair, Parinita Swarnkar, Keshav Swarnkar

**Affiliations:** 1 General Surgery, Aneurin Bevan University Health Board, Newport, GBR

**Keywords:** cancer colon, fit test, urgent suspected cancer, colonoscopy, ct

## Abstract

Aim: To assess recent colonoscopies and CT scans in conjunction with the feacal immunochemical test (FIT) for possibly downgrading urgent suspected cancer (USC) referrals.

Methods: A retrospective single-centre study was conducted, including all USC referrals for colonoscopy in 2022, excluding anal cancers. The CT and colonoscopy findings for a two-year period prior to the referral, along with the FIT result (if done), were noted. Combinations of tests were used to ascertain if any such combination would predict a negative colonoscopy (no cancer detected).

Results: Out of 500 USC referrals made, 160 were positive for colorectal cancer (CRC). Twelve cases had prior negative CT scans and colonoscopies, and none showed cancer (three were FIT and nine FIT not performed). A total of 54 cases had a prior CT with no FIT, four of which showed cancer. Fifteen cases had a prior CT with a negative FIT, and none showed cancer. Sixteen cases had prior negative colonoscopies, and all were negative for cancer (five negative FIT, one positive FIT, and 10 FIT not done ). Combining the categories where no cancer was missed, potentially 40 referrals could have been downgraded safely (8%).

Conclusions: The above data indicate that, among the patients being considered for a colorectal USC referral, a combined prior negative colonoscopy and CT scan warrants a downgrading of the referral (irrespective of the FIT result). Referrals with a prior negative CT (abdomen) with a negative FIT or normal colonoscopy within the last two years can be safely downgraded. This will have a positive impact on waiting times and monetary expenditure on the USC pathway, but larger studies would be required to prove the external validity of these findings.

## Introduction

An urgent suspected cancer (USC) referral is a request for an urgent patient appointment, typically from a general practitioner (GP), made based on symptoms and history indicative of a high suspicion of cancer [1]. Colorectal cancer (CRC) is the fourth most commonly diagnosed cancer in the UK; early diagnosis and excision of polyps can minimize the risk of malignant transformation.  Previous research has correlated shorter times to diagnosis for CRC with reduced mortality [2], highlighting the importance of early diagnosis and treatment.

The 2015 National Institute for Health and Care Excellence (NICE) guidelines widened the referral criteria for the two-week wait (TWW)/USC pathway for suspected lower gastrointestinal cancer. The 2017 (DG30 update) adoption of the quantitative faecal immunochemical test (FIT) for low-risk patients has proven to be a useful adjunct. These factors however have contributed to the volume of TWW referrals significantly increasing over 12 years, with nearly four times as many TWW referrals in 2020 (2,035) and 2019 (2,139) compared with 2009 (564), as per a Royal Surrey Trust study [3].

Among the other tools used for patients with high-risk symptoms of CRC, FIT performs well, with several large trials suggesting 87-94% sensitivity, 80-89% specificity, 12-18% positive predictive value (PPV), and 99% negative predictive value (NPV) at a threshold of 10 µg/g [4].

A study at Bedford Hospital including 125 patients who underwent both FIT and CT abdomen and pelvis (CTAP) prior to colonoscopy was conducted. Individually, FIT had shown similar results to other large trials. Performing FIT and CTAP in combination had a PPV and NPV of 28.6% and 100% respectively [5], which was superior to FIT alone.

From the above discussion, it is clear that there is a significant disease burden and early diagnosis of CRC is imperative for optimal results. Also optimizing the triage process for the urgent cancer pathway is essential to reduce waiting times for an already strained NHS to ensure continual delivery of high-quality care.

In this study, we attempt to see if combining the FIT test with previous known investigations (i.e., CT and colonoscopy) allowed safe downgrading of specific-criteria referrals from USC to urgent, thereby reducing the burden on an already strained USC pathway. 

## Materials and methods

This was a single-centre retrospective study including all patients identified as USC referrals (for colonoscopy) within the catchment area of our National Health Service Trust, Aneurin Bevan University Health Board (ABUHB), involving 500 patients, four hospitals, and a population of approximately 650,000.

Data were obtained from a centralized trust database, prospectively maintained for the National Bowel Cancer Audit (NBOCA). The cohort was selected by examining all the USC referrals on the trust endoscopy database to capture all relevant patients between 02-01-2022 and 29-09-2022 who had a colonoscopy in that time frame. Retrospectively, the project was approved by the hospital’s R & D department ensuring the maintenance of ethical standards and the protection of patient-sensitive data.

Patients with anal cancer were excluded from the study. All other USC referrals with an intention to identify colorectal cancer were included in the study. With the list of patients obtained from the endoscopy database, other details were obtained from the trusted software, which included individual demographics (age, sex), FIT results, and results from all investigations done in the last two years (CT abdomen pelvis - including CT colonograms/colonoscopies). An appropriate follow-up with histopathology was also done to confirm/rule out a malignancy after colonoscopy. Importantly, prior to the date of the USC colonoscopy, all investigations (i.e., previous colonoscopies, FIT, and CT scans) were checked for any suspicious findings. The primary outcome measure was NPV for each combination of tests to accurately predict the absence of cancer in the following two years.

The results were tabulated, and the NPVs of various combinations were assessed to see if they could be used as a reliable predictor of a negative colonoscopy (cancer-free colonoscopy). A demographic comparison was made to another large Welsh population-based study to ensure that the sample size was truly representative of the total population.

Definitions: USC, synonymous with the TWW pathway in England, describes patients that exhibit symptoms that are highly suspicious/showing red flag signs indicative of cancer. Such patients are aimed to have their first investigation within two weeks of the first point of contact with a medical professional.

Statistical analysis for the p-value was done using a chi-square test, with p<0.05 considered as being statistically significant. Statistical analysis was done with the assistance of the health board's statistician.

## Results

The study included 500 patients; 48.6% (n=243) female and 51.4% (n=257) male. The median age was 70 years (range: 19-96). The study cohort was compared with a similar study by Tang et al. [6] involving USC referrals in Wales, to prove that the data were not skewed, and there was no significant difference in the sample populations of the two studies (p-value for age and sex being 1 and 0.3, respectively). This added further credence to the fact that our sample was representative of the Welsh population (Table 1).

**Table 1:** Demographic comparison of data in a similar population of Wales. p value<0.05 is considered significant. Kindly refer to the reference section [6].

	Subramanian et al.	Tang et al. [6]	P value
Median age (yrs)	70	68	1
Sex ratio (Female:Male)	48.6:51.4	52.6:47.4	0.3

Out of the 500 USC referrals made, 160 were positive for CRC. We defined a negative computed tomography of the abdomen and pelvis (CTAP) as one that lacks any evidence of suspicious colonic thickening/mass, which would warrant a colonoscopy. A negative colonoscopy was defined as a colonoscopy, during which no more than one non-villous, non-proximal adenoma < 10 mm or serrated polyp < 10 mm was found. All prior investigations considered valid for evaluation were those conducted within two years of the USC referral date. The FIT threshold was taken as 10 µg/g. Levels above or equal to this were considered positive.

Twelve cases had prior negative CT scans and colonoscopies, all of which had no evidence of cancer after evaluation (of which three were FIT negative and nine FIT was not done). A total of 54 cases had a prior CT with no FIT, four of which showed evidence of cancer. Fifteen cases had a prior CT with a negative FIT, none of which showed cancer. Sixteen cases had prior negative colonoscopies, and all were found negative for cancer after evaluation (five of which had a negative FIT, one positive FIT, and in, 10 cases, the FIT test was not done) (Table 2).

**Table 2:** NPV of various investigative modalities and their combinations. NPV - Negative predictive value (excluding a colorectal malignancy). Parenthesis includes the number of cancer-negative cases/total cases. AP - Commuted tomography of the abdomen and pelvis. FIT - Faecal immunochemical test

	Combination of investigations	Negative predictive value (NPV)
1.	Prior negative CTAP and negative colonoscopy (irrespective of the FIT result)	100% (12/12)
2.	Prior negative CTAP with a negative FIT	100% (15/15)
3.	Prior negative colonoscopy	100% (16/16)
4.	Prior negative CTAP	92.6% (50/54)

Including only the subgroups with a 100% negative predictive value (combining prior negative colonoscopies and prior negative CTAPs with a negative FIT), we could justify downgrading 40 of the 500 referrals (8%) from a USC to an urgent referral.

## Discussion

According to Bowel Cancer Research UK, incidence rates have been stable for colon cancer since the 1990s, but the overall number of cancer cases per year is increasing [7]. There has been a disproportionate increase in USC referrals, which are associated with high costs. We sought to investigate screening modalities that are both clinically and financially effective.

This study has revealed that the following categories had a 100% NPV (provided the CTAP or colonoscopy was done in the preceding two years): combined negative FIT and previous negative CTAP, previous negative colonoscopy (irrespective of FIT), and combined previous negative CTAP and negative colonoscopy.

Among those with a combined negative FIT and previous negative CTAP, all 15 cases in our study showed no evidence of cancer, providing a reassuring 100% NPV. A study by Asmat et al. conducted at Bedford Hospital demonstrated FIT to have a PPV and NPV of 6.2% and 98.7%, respectively. When FIT was combined with a CTAP, the PPV and NPV rose to 28.7 and 100%, respectively [5]. This reiterates the fact that FIT, on its own, is a reasonable screening tool, but efficacy is improved by combining it with another modality.

Alternative options, such as CT colonography, are effective, especially with polyps above 10 mm with a sensitivity and specificity of 94 and 89%, respectively, with the advantage of being less invasive and more cost-effective. Unfortunately, there is an associated radiation exposure, and all abnormal tests would still need a colonoscopy [8]. Colvin et al. published that a CTAP had a 100% NPV in the detection of CRC when confirmed by colonoscopy (done within a year of the CT). However, the study did acknowledge the difficulty in the detection of polyps, especially those less than 10 mm [9]. Our findings justify the downgrading of such USC referrals with a combined negative FIT and negative CTAP, though larger multicentric trials would be necessary before it can be adopted as part of a structured guideline to prove its safety.

Among those with a previous negative colonoscopy (irrespective of FIT), our results demonstrated a 100% NPV (despite one patient having a positive FIT). This is in line with a study by Imperiale et al. at Indiana University USA involving 2,436 individuals with a normal baseline colonoscopy showing no cancer on the subsequent colonoscopy in five years [10].

Based on these findings, we hypothesize that combining colonoscopy results with the FIT results would serve no additional purpose. A study conducted at the University of California by Jain et al. comparing various modalities of colon cancer screening acknowledged the advantages of FIT, including low cost, easy accessibility, and high compliance. Colonoscopy remains important due to the low sensitivity of FIT; it allows for direct visualization and is both diagnostic, as well as therapeutic, resulting in the least frequent testing interval among the modalities available today. Unfortunately, the requirement of bowel preparation, interobserver variability, lower accessibility, and higher cost necessitate effective justification for using it as a screening modality. Other screening modalities, including colon capsule endoscopy, liquid biopsy methylated DNA, and stool-based microbiome tests, are being investigated for their efficacy and cost-effectiveness [8].

According to a Korean study by Jung et al., among patients who had a positive FIT with a colonoscopy within the last five years not needing a polypectomy, CRC was detected in only 0.72% of the population [11]. Another study by Rivero-Sánchez et al. conducted in Barcelona followed up 811 patients who had a negative colonoscopy after being FIT positive. They noted three post colonoscopy CRC (PCCRC) after 28 months of follow-up (0.4%). This reinforces the fact that a thorough colonoscopy is the most important factor in preventing PCCRCs [12]. A study in the USA involving 1,251,318 patients demonstrated that patients with a negative colonoscopy when followed up at two years showed a hazard ratio of 0.09 for developing CRC [13].

The combined BSG/ACPGBI post-polypectomy guidelines also indicate that even those individuals with high-risk features on colonoscopy (>=5 pre-malignant polyps or >=2 advanced colorectal polyps) will require a repeat colonoscopy in three years [14]. This guideline alone is a strong body of evidence to substantiate the downgrading of USC referrals with a negative colonoscopy in the UK.

Studies acknowledging missing cancer on colonoscopy are approximately between 2% and 6% [15]. We have not combined the FIT test along with previous colonoscopies as it appeared superfluous in this study. As larger multicentric studies are indicated, the relevance of FIT can be further studied to determine if it provides additional benefits in this category.

A previous standalone negative CT (without a FIT result) showed an NPV of 92.6%. This subgroup is a subset of the prior two categories. All the colonoscopies involved with this subgroup had a negative FIT.

We could not find any large study that combined prior colonoscopy and CTAP findings to assess the effect on subsequent CRC identification. Given accessibility to these modalities, a large multicentric study would be needed to formally assess the safety and feasibility of these recommendations.

A Lancet study in 2020 showed that a two-month delay in TWW investigatory referrals results in an estimated loss of between 0.0 and 0.7 life-years per referred patient, depending on age and tumour type, underlining the importance of preventing delays via this pathway [16]. In August of 2023, as per the official Bowel Cancer UK website, the monthly waiting times from NHS England saw 15% of patients waiting longer than two weeks to be seen by a bowel cancer specialist. However, in the same month, 43% of patients waited longer than 28 days to have bowel cancer ruled out or diagnosed [17], reflecting the pressure on the NHS to meet its targets.

In the UK, there are an estimated 41,700 new cases of CRC diagnosed each year. The number of endoscopy cases occurring per year in the UK is expected to increase from 1.7 million in 2015 to over 2.4 million in 2020. A diagnostic colonoscopy (considered the gold standard for diagnosis) costs £372, and therefore such a rise will cost the NHS approximately £250 million extra each year. Furthermore, current workforce availability means this rise would be unsustainable [18].

In a Welsh study by Tang et al. during the COVID period, they noticed that, of the 60 patients who were referred straight to test (colonoscopy/endoscopy), only seven (11.6%) showed evidence of CRC, and 34 (56.7%) were either normal or had benign pathologies [6]. This indicated a significant number of patients being referred straight for an endoscopy, a majority of whom did not have a sinister pathology. If these patients had an additional FIT, many unnecessary referrals could have been downgraded.

The new DG 56 guidelines of NICE recommend a USC referral for iron-deficiency anaemia without a FIT in two cohorts of patients: anyone aged over 60 or anyone aged over 50 years who has rectal bleeding [19]. It also states that clinical suspicion justifies a GP in making a USC referral; enabling GP access to prior records including colonoscopy reports and evidence-based guideline updates could stem low-risk referrals.

We concede that the small sample size could have resulted in bias, which reiterates the need for larger trials. Selection bias remains a limitation as health-conscious patients tend to present earlier and thus be investigated. The authors also acknowledge other limitations of the study, including occasional cases of grossly normal colonoscopies having missed small polyps; or in some cases, caecal intubation may have not been achieved (with respect to the prior colonoscopies) due to a multitude of reasons - most commonly patient discomfort or poor bowel preparation.

## Conclusions

The study identifies two potential subgroups (patients having a normal previous CT with a negative FIT and negative prior colonoscopy), which have shown a negative predictive value of 100% in our limited study. Therefore, we feel that USC referrals fitting these criteria can potentially be safely downgraded, resulting in a significant conservation of resources. We recommend a multicentric trial based on this study to assess its external validity and practical feasibility.
